# Genetic screening of Wnt signaling factors in advanced retinopathy of prematurity

**Published:** 2010-12-05

**Authors:** Miki Hiraoka, Hiroshi Takahashi, Hideo Orimo, Miina Hiraoka, Tsutomu Ogata, Noriyuki Azuma

**Affiliations:** 1 Department of Ophthalmology, Nippon Medical School, Tokyo, Japan; 2 Department of Biochemistry, Nippon Medical School, Tokyo, Japan; 3 Koganei Eye Clinic, Tokyo, Japan; 4 Endocrinology and Metabolism, National Research Institute for Child Health and Development, Tokyo, Japan; 5 Department of Ophthalmology, National Center for Child Health and Development, Tokyo, Japan

## Abstract

**Purpose:**

To evaluate the possibility of genetic involvement in retinopathy of prematurity (ROP). Although ROP is most often associated with low birthweight and low gestational age, these factors do not necessarily predict the severity of ROP. The possible involvement of other factors, including genetic variants, has been considered. Familial exudative vitreoretinopathy (FEVR) is a hereditary vitreoretinal disorder with clinical manifestations similar to those of ROP. Three genes involving the wingless/int1 (Wnt) receptor signaling pathway—*FZD4* for frizzled 4, *LRP5* for low-density lipoprotein receptor-related protein 5, and *ND* for Norrie disease protein—are associated with the development of FEVR.

**Methods:**

In the present study, 17 Japanese patients with advanced ROP were screened for these three candidate genes of FEVR. Genomic DNA from each patient was subjected to PCR and direct sequencing of the *ND*, *FZD4*, and *LRP5* genes.

**Results:**

One patient had a heterozygous mutation in the 5′ untranslated region of the *ND* gene. Another had a leucine insertion in the signal peptide of LRP5. None showed any mutation in *FZD4*.

**Conclusions:**

These findings suggest that genetic changes in the Wnt receptor signaling pathway associate to the development of advanced ROP.

## Introduction

Retinopathy of prematurity (ROP) is a vasoproliferative disorder of the eye affecting premature neonates and is a leading cause of visual loss in children even in developed countries. However, the pathogenesis of advanced ROP is not fully understood. It is known to occur in association with environmental factors, such as preterm gestational age and early oxygen exposure, but the same factors can lead to differing ROP phenotypes [1]. ROP regresses spontaneously in some patients but progresses rapidly to a severe stage in others. In addition, the incidence of advanced ROP varies among different ethnic groups [2]. These facts suggest that various internal factors, especially genetic variants, may be involved in the development of ROP [3].

Familial exudative vitreoretinopathy (FEVR) is a hereditary vitreoretinal disorder with clinical manifestations similar to those of ROP. FEVR is known to have three inheritance patterns: autosomal dominant (AD) [4]; autosomal recessive (AR) [5]; and X-linked recessive (XL) [6]. Four genes have been identified as candidates for FEVR: mutations of the Norrie disease (*ND*) gene have been found in XL-FEVR (the *ND* gene encodes the Norrie disease protein [NDP], Norrin) [7-9]; mutations of the *FZD4* gene encoding frizzled 4 have been observed in AD-FEVR [10,11]; and the *LRP5* gene encoding low-density lipoprotein receptor-related protein 5 (LRP5) is reported to show mutations in both AD-FEVR [11] and AR-FEVR [12]. Each molecule encoded by the above three genes participates in the wingless/integrated (Wnt) receptor signaling pathway. Norrin acts as a ligand in the Wnt receptor-β-catenin signaling transduction pathway by binding to frizzled 4 [13]. In the eye, Norrin plays roles in the recognition signal for targeting neuronal and retinal connections and in angiogenesis during retina development [14]. Frizzled 4 belongs to the frizzled family of Wnt receptors, and an *FZD4* knockout mouse model has demonstrated the importance of this pathway in angiogenesis and retinal development [10]. LRP5 is a member of the low-density lipoprotein receptor family. In Wnt signaling pathways, LRP5 forms a complex with frizzled 4, which acts as a functional receptor pair to activate the canonical Wnt-β-catenin pathway [15]. LRP5-deficient mice show a delay in hyaloid vessel regression, suggesting that LRP5 dysfunction can cause abnormal vascularization during retinal development [16]. Recently, another gene, *TSPAN12,* which is involved in the Norrin-β-catenin signaling pathway, has been found to be responsible for AD-FEVR [17-19]. Because of the resemblance between ROP and FEVR, genetic changes in the Wnt receptor signaling pathway during retinal development are considered to be likely risk factors for advanced ROP. This idea is supported by several recent studies involving screening for *ND* and *FZD4* gene mutations in ROP [20-25].

In the present study, we investigated whether the three genes responsible for FEVR are associated with advanced ROP and whether any association is related to ethnicity. This report discusses our findings regarding the Wnt signaling pathway and its effects on retinal development.

## Methods

### Patients

Seventeen premature infants (12 boys and 5 girls) were enrolled in this study via a protocol approved by the internal review boards of the National Center for Child Health and Development (Tokyo, Japan) and of the Nippon Medical School (Tokyo, Japan); informed consent was obtained from the parents of all of the subjects. Each patient was of a gestational age of less than 32 weeks, had a birthweight of less than 1,500 g, had undergone eye surgery, and had retinal findings consistent with advanced ROP (Table 1). The stage of ROP was determined by trained ophthalmologists according to the international classification of retinopathy of prematurity [26].

**Table 1:** List of patients enrolled in this study.

**Case number**	**Sex**	**Gestational age (week)**	**Birthweight (g)**	**Stage of ROP right/left**	**Type of ROP**	**Surgery received**	**Operation**
1	M	27	1,084	4A/4A	classic ROP	both eyes	buckling
2	M	25	972	5/5	AP-ROP	both eyes	vitrectomy
3	F	26	906	4A/4A	AP-ROP	both eyes	vitrectomy
4	M	23	596	4A/4A	AP-ROP	both eyes	vitrectomy
5	F	29	1212	4A/3	AP-ROP	right eye	vitrectomy
6	M	23	512	5/5	AP-ROP	both eyes	vitrectomy
7	F	23	642	5/5	AP-ROP	both eyes	vitrectomy
8	M	24	670	4A/5	AP-ROP	both eyes	vitrectomy
9	M	23	542	5/5	AP-ROP	both eyes	vitrectomy
10	F	24	588	4A/3	AP-ROP	both eyes	vitrectomy
11	M	23	747	4A/3	AP-ROP	right eye	vitrectomy
12	M	27	998	4A/4A	AP-ROP	both eyes	vitrectomy
13	M	23	458	5/5	AP-ROP	both eyes	vitrectomy
14	M	23	560	4B/4A	AP-ROP	both eyes	vitrectomy
15	M	23	676	5/3	classic ROP	right eye	vitrectomy
16	F	23	708	5/5	AP-ROP	both eyes	vitrectomy
17	M	24	576	5/5	AP-ROP	both eyes	vitrectomy

Stage of ROP was classified based on ICRP. AP-ROP; aggressive posterior ROP.

### Genetic analysis

Genomic DNA was isolated from peripheral blood and amplified with PCR. Each pair of oligonucleotide primers is provided in Table 2 and Table 3. To design the primers and identify the gene variations, we used reference complementary cDNA sequences of *ND* (NM 000266.3), *FZD4* (NM 012193.1), and *LRP5* (NM 002335.1) obtained from GenBank (National Center for Biotechnology Information). The *ND* gene had three exons, and three pairs of primers were used to amplify each exon. The primer pairs for exons 1 and 2 covered the 5′ untranslated region (UTR) and coding region, and the primer pair for exon 3 amplified the coding region. An *FZD4* gene with two coding regions was amplified with seven primers (Table 2). Primers 1A forward and 1B reverse were used to detect exon 1, including the 5′ UTR and coding region. The internal primer BF was used to detect the coding region. Exon 2 had a long coding region so it was amplified in two overlapping segments, A and B. *LRP5* consisted of 23 exons and encoded 1,615 amino acids. Each exon was amplified with the primer pairs listed in Table 3. The primer pair for exon 1 amplified the coding region and part of the 5′ UTR. Two pairs were used to detect exon 23 including the 3′ UTR.

**Table 2:** Primer sequence for *ND* and *FZD4* gene amplification.

**Name**	**Primer sequence (5′> 3′)**	**Product size (bp)**
*NDP*-1	F: CGCCTGATTGATATATGACTGCAATGGC	322
	R: GCTCGGTTTGGAAAGAAGCGATTTCCT	
*NDP*-2	F: TTCTGGGTAAATAATTCTGGGG	471
	R: GTTTCTGAGGGAAATGCTCTCCTCACA	
*NDP*-3	F: TAAGGTTGTGGCATGCCCACAGAGTAA	690
	R: CAGAAGATGTCCCAGGAAAAGCTGGGCTTT	
*FZD4*-1A	F: ATAATTTTAGCGCCGCGAGCCTCCAG	771
	R: GAAATCACTTTTCCAGGAGAGCTGTCTCC	
*FZD4*-1B	F: CAAACTGGGGGTGTCTGCCAGAGCA	475
	R: GAAATCACTTTTCCAGGAGAGCTGTCTCC	
*FZD4*-2A	F: GGGAGCATTTGGTCAAACTTCCAAGTC	757
	R: GAGTGTCAGAATAACCCACCAAATGGAGCT	
*FZD4*-2B	F: CCTGTTCTCATCCAAGAAGGACTTAAGAA	781
	R: TTCAAAATGAAGAAAGCATGGAGGCTGACT	

**Table 3:** Primer Sequence for *LRP5* gene amplification.

**Name**	**Forward sequence (5′→3′)**	**Reverse Sequence (5′→3′)**	**Product size (bp)**
Exon 1	TCCTCCCCGTCGTCCTG	ATTGTCCGAGCAACCCG	269
Exon 2	CTTAGCCAGTGGCCCTCA	AGAGAGAGATGGTGACACT	496
Exon 3	TCTGTGTTAGCTGCTTCTCTT	CCAGGACTGCGTGGGTA	259
Exon 4**	GATGGCTCCTCCACCCCGCT	GCGCCCCAGCCGGCACT	250
Exon 5**	CTCATTCAGAAACAAGTGACGGTCCTC	GTCCCGTCCCACCGCCT	216
Exon 6	CTGCTGCAGGCCCTTGA	TCTCCCTCTCGCCTGTG	506
Exon 7	GTCATGGACTTCTGCTTCTT	TGGCCTCCTGGATCAAAC	235
Exon 8	TGGCCCATCCAGACCTAT	CAAGTCTGCATGGCTGAG	298
Exon 9	GCATTCATTGTGTGGCTTG	AAGCCTTTGAGGCAGGA	446
Exon 10	CTTTTCCTCCTCACCTGCT	GGTGAACACAAGGACGC	289
Exon 11	AGACTCACTGAGCCTGC	GCCCTCCATGACCAGAAG	261
Exon 12	CCTTTGCTGACACCGTG	GAAGCTCCTTTCAGCGT	440
Exon 13	CCTGCAGCCCTGTCTTT	GCCTTGGGAAGCACACC	271
Exon 14	CTCAGGAGTCTTGGTTTCTTT	GCATTCGGCAGAAGACAC	308
Exon 15	CCCACACCCGTCCTTCA	GGGTGTCTGCGGTTAGG	263
Exon 16	GAGGTCAGCACTGCTCA	GGTCGGGTTTAGAGGCCA	301
Exon 17	AGAGCCTGACCTCTGTTT	TACCTGTCCATCACCCCAA	206
Exon 18	GGCTGCGTGTGATGTTC	GGTCTTGGCAGAGCCTTGA	307
Exon 19	AGCCTCTCTGAGTGCAT	TAAACTCCACGTTCCTGGG	208
Exon 20	GGCCACCTCTTTCTGTTT	AGATCATTCCATATCTCAGGCTC	294
Exon 21**	GAGTCTCGTGGGTAGTGGGA	AGAAAGCAAGGCATGCCTCAGAG	245
Exon 22	CTGGCGAGGCTCTAAGT	CCCAATGGCCATGGAGG	174
Exon 23A*	CTCCTCTGTGTGTGTCCC	TCAGGATGAGTCCGTGC	286
Exon 23B*	CTACTTCCATCTCTTCCCGC	CGAAAGAATGGCAGTTCTGTT	271

Primer sequences obtained from Gong et al. [16] ** Exon 23 was amplified in two overlapping segments, A and B.

PCR was performed with LA Taq polymerase (Takara Bio, Otsu, Japan) according to the manufacturer’s recommended protocol. For *ND* and the A set of *FZD4*, 10X LA PCR™ buffer II (Takara Bio) was used. GC buffer I (Takara Bio) was used for the other primer pairs. The amplification conditions for *ND* and *FZD4* involved 30 cycles of denaturation at 98 °C for 30 s, annealing at 58 °C for 30 s, and extension at 72 °C for 1 min. For *LRP5*, amplification was performed using 30 cycles of denaturation for 30 s at 98 °C, followed by 30 s at 61 °C. The PCR products were purified with a PCR clean-up kit (Macherey-Nagel, Düren, Germany) and sequenced with BigDye^TM^ Terminator version 3.1 (Applied Biosystems, Foster city, CA) on an ABI PRISM® 310 Genetic Analyzer (Applied Biosystems).

## Results

Of the 17 infants, 12 were boys and five were girls. Birthweights ranged from 458 to 1,212 g with a mean (±standard deviation [SD]) of 732±221 g; gestational ages ranged from 23 to 29 weeks with a mean (±SD) of 24.3±1.9 weeks. Of the cases of ROP in the patients' 34 eyes, four were classified as ROP stage 3, 13 as stage 4A, one as stage 4B, and 16 as stage 5. Fifteen of the patients showed aggressive posterior (AP) ROP, and the others had classical ROP. Fourteen had undergone surgery on both eyes, and the other three on one eye. Vitrectomy had been performed on 16 eyes and buckling on one eye (Table 1).

**Figure 1:**
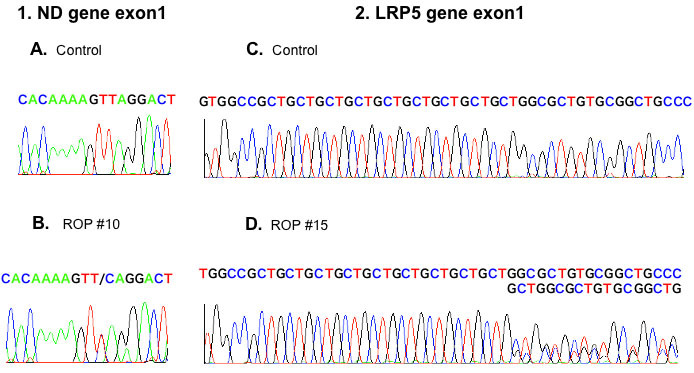
Sequence chromatograms of mutations in retinopathy of prematurity. Left: The chromatogram of *ND* gene exon 1. It shows the antisense sequence between nucleotide 231 and 245. Retinopathy of prematurity (ROP) #10 shows a heterozygous mutation at 237 T->C. Right: The chromatogram of *LRP5* gene exon 1. **C**, **D** indicate a sense sequence including CTG repeat regions. The text below each normal chromatogram represents a mutated sequence. ROP #15 shows a heterozygous 3-bp insertion (CTG) that introduced another leucine insertion in the polyleucine region. **A**, **C** indicate a sequence of an unaffected individual. **B** indicates a sequence of ROP #10. **D** indicates sequence of ROP #15.

Sequencing of three genes was performed. On *ND* gene screening, one patient (#10) showed a heterozygous A>G mutation in the 5′ UTR of exon 1 at position 237. This mutation was not found in 51 unaffected Japanese subjects. In one patient (#15) *LRP5* gene analysis revealed a heterozygous 3-bp insertion within the CTG repeat region of exon 1, which introduced another leucine insertion in the polyleucine region within the signal peptide (Figure 1). This insertion was confirmed with antisense sequence (data not shown). This insertion was not found in 28 unaffected Japanese subjects. No *FZD4* mutations were found in any of the patients.

## Discussion

A genetic involvement in advanced ROP has been suspected for a decade. In the present study we performed genetic screening of three molecules involved in the Wnt signaling pathway in 17 Japanese infants with advanced ROP (screening patients with uniform ethnic backgrounds is advantageous because genetic polymorphisms due to racial differences can be avoided). One patient had a point mutation in the 5′ UTR of the *ND* gene, and another had a 3-bp insertion resulting in one amino acid insertion in the signal peptide of the *LRP5* gene. These findings suggest an association between genetic factors and the development of ROP.

The three genes chosen for investigation in this study are responsible for FEVR and are involved in retinal development through the Wnt signaling pathway. The *ND* gene mutation that causes XL-FEVR has been identified. Because the incidence of severe ROP is higher among males than among females, the abnormality of the X chromosome, which includes the *ND* gene, might be one of factors that influences the severity of ROP [27]. Several studies have demonstrated *ND* gene variants in ROP [20-22]. In this study, one patient showed a heterozygous single base-pair alternation in the 5′ UTR at position 237 with an A to G nucleotide change. Because the 5′ UTR is responsible for transcription regulation and translation efficiency, this mutation may have altered gene regulation. An inability to activate the Wnt receptor-β-catenin pathway results in the early abrogation of neurosensory and vascular development [14]. Several reports have described genetic variants in the 5′ and 3′ UTRs of the ND gene in patients with ROP, but most mutations have been detected within the coding region in patients with Norrie disease [28]. ROP and Norrie disease have different phenotypes and time of onset as well as speed of disease progression; while Norrie disease causes bilateral retinal malformation, mental retardation, and deafness, the main features of ROP are abnormal retinal vascular development. It has been speculated that incomplete ND proteins with amino acid changes result in malformation of the ocular tissues, whereas inappropriate gene expression affects retinal vascular conformation.

Several reports have referred to the role of the *FZD4* gene in ROP [23-25]. Four missense mutations have been reported in severe ROP. Extensive screening of the 5′ UTR and coding region in our study failed to detect any genetic variants, and further investigation is required to clarify the role of the *FZD4* gene in advanced ROP.

Although several mutations of *LRP5* have been found in FEVR, this study is, to our knowledge, the first to perform genetic screening in ROP. We screened the entire coding region and 3′ UTR of *LRP5*. Due to the presence of a pseudogene on chromosome 22 that has high homology with the *LRP5* 5′ UTR, it is difficult to isolate the entire 5′ UTR with PCR [16]. We detected a heterozygous polymorphism in one patient—a 3-bp insertion at the CTG repeat region in exon 1, leading to the addition of a leucine amino acid to the polyleucine residue within the signal peptide. Therefore, the genetic variant in our case with the insertion of a single leucine in the signal peptide might lead to insufficient translocation during protein processing and affected retinal development. In functional assays for the LRP5 signal peptide variants, one leucine insertion in the polyleucine residue impairs Norrin signal transduction [29]. The same group Chung et al. [29] has identified that approximately 10% of German and Turkish unaffected subjects have a heterozygous allele with 10 leucines in the LRP5 signal peptide residue. In contrast, none of the 28 unaffected subjects had this insertion in our study. This may be due to ethnic background differences. *PCSK9*, a gene implicated in cholesterol metabolism, is known to have polymorphism within signal peptide polyleucine stretches among normal subjects. The heterozygous carriers of a 10-leucine allele had lower low-density lipoprotein cholesterol concentration compared to homozygous carriers of a 9-leucine allele [30]. Taken together, we speculate that the genetic change in our case may have influenced retinal development under premature circumstances.

In conclusion, through analysis of genes involving the Wnt receptor signal pathway, we have identified two genetic variants in advanced ROP. Our results suggest that abnormality in the Wnt signaling pathway during retinal development may associate to severe ROP. Although the mutation and polymorphism are implicated in a small number of cases, the risk factors for advanced ROP might be polygenetic. Additionally, the comparison of genetic change between severe and mild cases can help clarify the etiology of ROP; however, a mild case sample was not available to us. Unfortunately, we are unable to determine whether these genetic changes are de novo or inherited because the parents declined to be tested.

Extensive genetic analysis with an increased sample number and genes, including *TSPAN12*, should lead to a better understanding of the pathogenesis of ROP.
